# T cell therapy in combination with Vemurafenib in BRAF mutated metastatic melanoma patients

**DOI:** 10.1186/2051-1426-2-S3-P67

**Published:** 2014-11-06

**Authors:** Troels Holz Borch, Rikke Andersen, Per Kongsted, Özcan Met, Mads Hald Andersen, Per thor Straten, Marco Donia, Inge Marie Svane

**Affiliations:** 1 Center for Cancer Immunotherapy, Herlev, Denmark; 2 Center for Cancer Immune Therapy, Dept. of Hematology and Dept. of Oncology, Copenhagen University Hospital, Herlev, Denmark, Herlev, Denmark

## Background

Adoptive T cell therapy (ACT) with tumor infiltrating lymphocytes (TIL) has proven to be a powerful treatment option for patients with metastatic melanoma, even when heavily pretreated, with response rates of approximately 50% and durable complete responses in about 15%. However, there is still a need for improving TIL efficacy and a promising strategy is combination with immunomodulating agents. One such is Vemurafenib (Vem), a selective BRAF inhibitor, which has been shown to induce objective responses in about 50% of treated melanoma patients expressing BRAF^V600E/K^, improving progression-free survival and overall survival compared to standard chemotherapy. In addition to the direct anti-cancer effect, Vem has been shown to increase T cell infiltration into tumors, up-regulate melanoma antigen expression and increase the frequency of TIL recognizing autologous melanoma cells. Combining TIL treatment and Vem is currently being investigated in other clinical trials testing different treatment schedules (http://ClinicalTrials.gov Identifier: NCT01659151 and NCT01585415).

## Methods

A total of 12 patients will be included in this Phase II trial primarily to investigate safety when combining ACT and Vem. Secondarily, clinical responses will be evaluated according to RECIST and extensive immune monitoring will be performed.

Patients will receive Vem orally 960 mg BID one week prior to excision of tumor material for T cell generation and continue this treatment until hospital admission. The patients will be hospitalized one week prior to TIL infusion in order to follow a preparative lymphodepleting regimen consisting of Cyclophosphamide 60 mg/kg for 2 days and Fludarabine 25 mg/m^2 ^for 5 days. TIL infusion typically consists of 5-10 × 10^10 ^T cells and patients are subsequently treated with continuous interleukin-2 infusion following the decrescendo-regimen for 5 days.

Patients will be evaluated 6 weeks after TIL infusion and continuously thereafter.

## Results

Patient accrual will start September 2014.

